# Bone Marrow Cells in Acute Lymphoblastic Leukemia Create a Proinflammatory Microenvironment Influencing Normal Hematopoietic Differentiation Fates

**DOI:** 10.1155/2015/386165

**Published:** 2015-05-18

**Authors:** Armando Vilchis-Ordoñez, Adriana Contreras-Quiroz, Eduardo Vadillo, Elisa Dorantes-Acosta, Alfonso Reyes-López, Henry Martin Quintela-Nuñez del Prado, Jorge Venegas-Vázquez, Hector Mayani, Vianney Ortiz-Navarrete, Briceida López-Martínez, Rosana Pelayo

**Affiliations:** ^1^“Federico Gómez” Children's Hospital, 06720 Mexico City, DF, Mexico; ^2^Oncology Research Unit, Oncology Hospital, Mexican Institute for Social Security, Avenida Cuauhtemoc 330, Colonia Doctores, 06720 Mexico City, DF, Mexico; ^3^Clinical Biochemistry Program, National Autonomous University of Mexico, 04510 Mexico City, DF, Mexico; ^4^Molecular Biomedicine Department, CINVESTAV, 07360 Mexico City, DF, Mexico; ^5^UMAE “Dr. Victorio de la Fuente Narvaéz”, Mexican Institute for Social Security, 07760 Mexico City, DF, Mexico

## Abstract

B-cell acute lymphoblastic leukemia (B-ALL) is a serious public health problem in the pediatric population worldwide, contributing to 85% of deaths from childhood cancers. Understanding the biology of the disease is crucial for its clinical management and the development of therapeutic strategies. In line with that observed in other malignancies, chronic inflammation may contribute to a tumor microenvironment resulting in the damage of normal processes, concomitant to development and maintenance of neoplastic cells. We report here that hematopoietic cells from bone marrow B-ALL have the ability to produce proinflammatory and growth factors, including TNF*α*, IL-1*β*, IL-12, and GM-CSF that stimulate proliferation and differentiation of normal stem and progenitor cells. Our findings suggest an apparently distinct CD13^+^CD33^+^ population of leukemic cells contributing to a proinflammatory microenvironment that may be detrimental to long-term normal hematopoiesis within B-ALL bone marrow.

## 1. Introduction

Inflammatory cells and their products are important regulators of the tumor microenvironment. Based on experimental models and epidemiological data, increasing evidence has suggested the close connection between chronic inflammation and carcinogenesis, particularly in colorectal cancer, gastric cancer, and liver and lung neoplastic diseases [1–3]. Moreover, it has been estimated that about 25% of tumors are associated with chronic inflammatory processes caused by infections or unknown causes [1]. Inflammation may associate with cancer through two potentially connected pathways: the extrinsic, which results from external factors promoting latent inflammatory responses, and the intrinsic, conducted by oncogenes or tumor suppressor genes that activate the expression of inflammation-related programs [4]. For both of them, the cellular and cytochemical components within the tumor microenvironment are crucial. Macrophages are the most widely described inflammatory cells related to cancer [5, 6], while tumor-associated fibroblasts have been identified as good producers of proinflammatory cytokines and growth factors in skin, breast, and pancreas malignancies [7]. The participation of cells of the immune response in the initiation, growth, and tumor progression, as well as in the response to antineoplastic therapy, is apparently mediated by a variety of proinflammatory cytokines, including IL-1*β*, IL-1*α*, TNF*α*, IL-6, IL-12, and IFN*γ*, and chemokines as CCL2 and CXCL12 [8, 9]. They play a role in tumorigenesis and tumor-associated inflammation has made them potential targets for adjuvant therapy in cancer. Of special interest, transcription factors such as NF*κ*B and STAT3 may cooperate to promote the development and progression of cancer, by controlling the expression of antiapoptotic and proliferative genes, in addition to intervening in the regulation of angiogenesis and invasion [1, 8, 10]. Accordingly, the TLR-MyD88 signaling pathway has been shown to promote tumorigenesis in primary carcinogen models. Thus, together, cytokines, chemokines, and their receptors are keys to the binomial cancer-inflammation entity, compromising sometimes the physiological functions and promoting normal cell senescence, concomitant to proliferation and survival of tumor cells with invasive and metastatic properties [9, 11].

Despite the increasing evidence of anomalies in the bone marrow microenvironment of hematologic malignancies, which may govern stem cell activity and lead to disease (reviewed in [12, 13]), knowledge about the role of inflammation in leukemogenesis is scarce. At present, B-cell acute lymphoblastic leukemia is the most common cause of cancer in children worldwide. It is characterized by uncontrolled production of hematopoietic B-precursor cells within bone marrow (BM), with inexorable damage to their differentiation and functional properties. Although a number of genetic aberrations potentially contribute to leukemogenesis, the cellular origin of B-ALL is a fundamental issue under research [12, 14, 15]. Both, cell culture systems and animal models of human-mouse xenotransplantation and leukemic reconstitution suggest that leukemia-initiating cells have characteristics of primitive progenitors [16] and are sensitive to instability lineage triggered by extrinsic stimuli [13, 17]. According to the original hypothesis of leukemogenesis of Greaves, the occurrence of multiple consecutive lesions in hematopoietic cells can trigger malignant transformation, opening the possibility that not only the oncogenic damage inherent in the early stages of development, but abnormal microenvironmental factors could contribute directly or indirectly to generation or maintenance of leukemic precursors [18]. The implication of specific genetic aberrations in the triggering of ALL-inflammation setting will be highly relevant to investigate. A role of the fusion gene ETV/RUNX1 TEL/AML1 in the TGF signaling constitutes a milestone entailing this immune regulatory pathway to leukemogenesis, by compromising the normal cell responses to its inhibitory function. Accordingly, the favoring of reactive oxygen species by ETV6/RUNX1 has been recently reported that apparently origins in the B-cell progenitor compartment [19–21]. Furthermore, the competition of leukemic cells with normal hematopoietic cells by the normal marrow niches and their apparent contribution to formation of abnormal inhibitory microenvironments have suggested the exhaustion or migration of normal precursors, resulting in almost total BM replacement by malignant cells [22–24]. Remarkably, B-ALL primitive cells before and upon cultures have been shown to produce high levels of TNF*α* and IL-6, suggesting that an inflammatory microenvironment prevails in this BM disorder ([25, 26] and our unpublished observations). More recently, the studies from Guzman et al. indicate that leukemic stem cells in acute myeloid leukemia have constitutively activated the transcription factor NF*κ*B [27, 28], a fact that may, in turn, harm the normal biology of hematopoietic niches. To determine whether lymphoblastic leukemia precursors contribute to an inflammatory BM microenvironment that may impact the earliest hematopoietic development from the onset of the disease, we have now investigated and confirmed the production of soluble factors, including cytokines, chemokines, and growth factors by mononuclear cells from B-ALL BM. By using* in vitro* proliferation assays and controlled culture systems, the impact of ALL-derived proinflammatory factors on normal hematopoietic differentiation potentials was examined, suggesting that within ALL marrow, normal primitive cells are driven into cycle and prompted to produce both lymphoid and innate lineage cells.

## 2. Materials and Methods

### 2.1. Patient Characteristics and Sample Collection

Fifty-four children referred to the “Federico Gomez” Children's Hospital (Mexico City, Mexico) and diagnosed with B-cell precursor acute lymphoblastic leukemia were included in the study. Among them, 34 patients fulfilled the criteria for high-risk disease by blood cell count, age, T-cell phenotype, or Ph+ chromosome, whereas 17 for fulfilled the criteria standard-risk. Within the high-risk group, 35% of the patients were female and 65% were male, while the standard risk group included 24% female and 76% male patients. The median age values were 7.8 year old (2 mo–18 yr) and 4.8 (2 yr–9 yr) for the high-risk and standard-risk group, respectively. BM specimens were collected by aspiration before any treatment, respecting international and institutional guidelines. Control BM specimens were obtained from healthy children undergoing minor orthopedic surgery. All procedures were approved by the Ethics, Research and Biosafety Committee of the “Federico Gómez” Children's Hospital (Registry HIM/018/2013) in Mexico City. Umbilical cord blood (UCB) samples were obtained from normal full-term neonates. All samples were collected after informed consent from the parents.

### 2.2. ALL Phenotyping and Pattern Definition

Patients fulfilling morphological criteria of ALL according to the French-American-British group (FAB) were stratified in line with clinical risk of relapse and phenotypic expression of CD10, CD19, CD20, CD22, CD79*α*, Kappa, Lambda, anti TdT, CD3, CD5, CD7, CD13, CD14, CD15, CD33, myeloperoxidase, CD45, and CD34. Patient groups were further defined in accordance with aberrant expression of myeloid markers, with 25% of the cells expressing the myeloid antigen being considered as positive. Cytokine production based on the expression of two or more antigens was assessed in ALL.

### 2.3. Cell Isolation and Supernatant Collection

Mononuclear cells (MNC) from B-cell precursor ALL patients were prepared by Ficoll-Paque Plus (GE Healthcare Bioscience) gradient separation and placed in culture with Alpha MEM medium (Gibco by Life Technologies) (200,000 MNC per 200 *μ*L medium). Supernatants were collected at 24 h and evaluated for cytokine concentration (experimental time = 24 h). Additional 200 *μ*L of culture medium was added to the same cells and their supernatants were reevaluated 24 h later (experimental time = 48 h).

### 2.4. Cytokine Detection

Supernatants were collected and stored at −20°C until analysis. The cytokine, chemokine, and growth factor content in supernatants were measured by Multiplex Milliplex Map Immunoassay (Merck Millipore), following the manufacturer's recommended protocols. The assay included SDF-1, BCA-1, LIF, TRAIL, SCF, TSLP, FGF-2, Flt-3L, G-CSF, GM-CSF, IFN*α*, IFN*γ*, IL-1*α*, IL-1*β*, IL-2, IL-3, IL-4, IL-5, IL-7, IL-8, IL-10, IL-12(p40), IL-12(p70), IL-15, IP-10, TNF*α*, and VEGF.

### 2.5. Immunofluorescence Microscopy

ALL and control MNC were fixed using 4% paraformaldehyde and hydrated with PBS. Permeabilization was performed with 0.01% Triton X-100 (Bio-Rad) and blocking with 2% BSA for 30 minutes at 37°C. Anti-pNF*κ*B p65 (serine 536) and anti-pSTAT3 (tyrosine 705) antibodies (Cell Signaling) were incubated for 1 hour at 37°C followed by incubation with goat anti-rabbit Alexa 488 (Invitrogen) antibody. Slides were mounted with Vectashield with propidium iodide (Vector). Overlay of images from the different fluorescent channels was performed using ImageJ software (NHI) and Image Pro Plus software.

### 2.6. Purification of Precursor Cells

Mononuclear cells (MNC) from umbilical cord blood (UCB) were prepared by Ficoll-Paque Plus (GE Healthcare Bioscience) gradient separation. CD34^+^ cells containing hematopoietic stem and progenitor cells were enriched from the MNC fraction using the Human CD34 Progenitor Cell Isolation Kit (Miltenyi Biotec) according to manufacturer instructions, with final purity frequencies between 85 and 95%. Cells were enumerated before analyses, and the purity confirmed by flow cytometry.

### 2.7. CFSE Proliferation Assay

Carboxyfluorescein diacetate succinimidyl ester (CFSE) was used for* in vitro* labeling of cells to trace multiple generations using dye dilution by flow cytometry (Molecular Probes). CD34^+^ CB cells were incubated with 10 mM CFSE and then exposed in a 120-hour culture to supernatants collected from control BM MNC (Control SN), noninflammatory BM MNC (Non Infl SN), or inflammatory BM MNC (Infl SN). After the 5 days, harvested cells were analyzed by flow cytometry for their phenotype and the number of cell divisions. Dilution of fluorescence intensity to monitoring up to 8 cell divisions was estimated using the application for cell proliferation within the FlowJo 7.6.1 software.

### 2.8. Stromal Cell Co-Cultures

UCB precursor cells were placed on MS-5 stromal cell monolayers and cocultured with them for 3 weeks in the presence of ALL MNC supernatants and with lymphoid conditions, according to a modified previous report [26]. The *α*-modified essential medium (*α*-MEM) was supplemented with 10% fetal bovine serum, 1 ng/mL Flt3-L (FL), 2 ng/mL SCF, 5 ng/mL IL-7, and 10 ng/mL IL-15 (Preprotech) and contained 100 U/mL penicillin and 100 mg/mL streptomycin. Supernatants collected from control BM MNC (Control SN), noninflammatory BM MNC (Non Infl SN), or inflammatory BM MNC (Infl SN) were used in a 3 : 1 medium : supernatant volume ratio. Coculture systems were incubated at 37°C in a humidified atmosphere of 5% CO_2_. Media and supernatants were weekly replaced. This controlled system promotes the efficient differentiation of hematopoietic stem, progenitor, and precursor cells towards B cells, NK cells, lymphoid-related dendritic cells, and some myeloid cells. Cell frequencies and final yield per input values were calculated on the basis of specific lineage production within each condition.

### 2.9. Proliferation and Differentiation Analyses by Flow Cytometry

Phenotyping of UCB hematopoietic cells from lymphoid cocultures was performed by six-color flow cytometry on a FACSCanto flow cytometer (BD Biosciences). Cells were enumerated after culture to calculate cell frequencies and yields per input progenitor prior to staining with directly conjugated fluorescent antibodies (Invitrogen and BD Pharmingen). For the CFSE proliferation assay, fluorochrome-conjugated antibodies anti-lineage markers (CD3, CD8, TCR, CD56, CD14, CD11b, CD20, CD19, and CD235a) and anti-CD34 were used to identify the Lin^−^CD34^+^, Lin^−^CD34^−^, and Lin^+^CD34^−^ populations. On the other hand, at the end of the differentiation cultures, harvested cells were stained with antibodies anti-CD34, -CD56, -CD11c, -CD19, -CD11b, and -CD14, and newly produced cells were identified by their phenotype as follows: stem and progenitor cell fraction as CD34^+^; NK precursor cells as CD34^+^CD56^+^ cells; NK cells as CD34^−^CD56^+^ cells; dendritic cells as CD11c^+^, and B cells as CD19^+^, whereas myeloid cells were CD11b^+^CD14^−^ or CD11b^+^CD14^+^ cells. Analysis of flow cytometry data was performed using the FlowJo 7.6.1 software, and final yield per input values was calculated on the basis of specific lineage cell frequencies within each condition.

### 2.10. Statistics

The Prism (version 5.01, GraphPad) and SPSS software were used for statistical analysis. Comparisons between groups were performed with the unpaired *t*-test. *P* values were two-tailed and were considered significant if less than 0.05. Additionally, for the aberrant expression of myeloid markers, a distributional analysis of the data was made and found no normal distribution. Thus, comparison groups were performed with the nonparametric test *U* Mann-Whitney, comparing the medians and taking *α* of 5% to define statistical significance.

## 3. Results and Discussion

### 3.1. Two Groups of B-ALL Patients according to BM Hematopoietic Cell Cytokine Production

The hematopoietic microenvironment within bone marrow (BM) is constituted by a cellular network and its products (including extracellular matrix, cytokines, chemokines, and growth factors), which form a highly organized three-dimensional structure to support hematopoiesis [12, 29]. Under normal conditions, the current model of hematopoietic microenvironment includes at least two specific cell niches, according to which stem cells require interaction with osteoblasts and endothelial cells, whereas the earliest progenitors are dependent on the contact with stromal cells expressing CXCL12/SDF1, and downstream lineage committed precursors of B cells require IL-7. The recent discovery of regulation of the hematopoietic developmental pathways by pathogen and/or danger recognition by primitive cells suggests that Toll-like receptors (TLR) are involved in the early cell fate decisions and contribute to the emergent replenishment of innate hematopoietic cells in the context of inflammatory settings [13, 30–38]. Moreover, the production of proinflammatory cytokines and growth factors, including TNF*α*, IL-12, IL-3, IL-6, IL-8, GM-CSF, and MCP-1, as a result of TLR signaling in normal progenitor cells, has been documented [37, 39–41]. In response to these inflammatory signals, hematopoietic stem and early progenitors can, in turn, produce cytokines that promote their mobilization and expedite differentiation to innate cells [42, 43].

**Figure 1:**
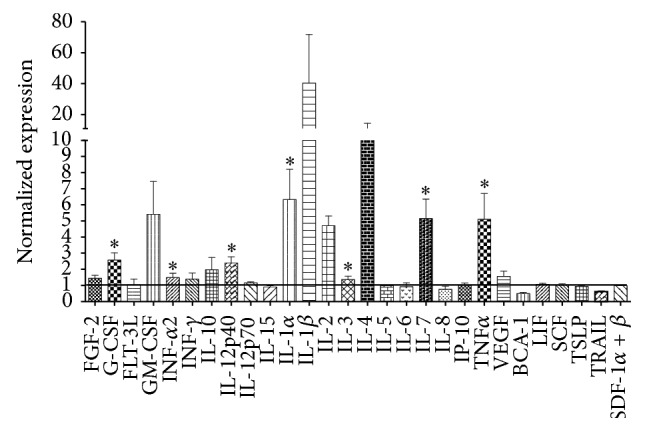
Bone marrow cells from childhood acute lymphoblastic leukemia produce proinflammatory cytokines and hematopoietic growth factors. ALL mononuclear cells from BM aspirates were cultured for 24 h, and supernatants were further collected and assayed for 28 cytokines, chemokines, and growth factors. Normalized production relative to normal bone marrow cells was tabulated.

Derived from the tumor microenvironment, leukemic cells have been shown to harm normal biology of hematopoietic cells [22]. Therefore, cytokines and growth factors were now quantified in supernatants derived from B-ALL MNC. Remarkably, the proinflammatory factors IL-1*α*, IL-1*β*, and TNF*α* were highly overproduced when compared to production levels by their normal counterpart, with up to 40-time increases for IL-1*β* (Figure 1). Moreover, some cytokines, interferons, and growth factors participating in inflammatory responses, including G-CSF, GM-CSF, IFN*α*, and IL-12 were substantially elevated in B-ALL. The same was true for early lymphoid-related growth factors, like IL-7 (Figure 1). Thus, for the first time, hematopoietic precursors in childhood B-cell acute lymphoblastic leukemia have been shown to produce proinflammatory and hematopoietic growth soluble factors. Modest increments were observed in IFN*γ*, IL-10, and VEGF, while no changes were registered in Flt3L, IL-15, IL-5, IL-6, IL-8, SCF, and SDF-1. Accordingly, although the evaluation of cytokines and growth factors in patients with acute lymphoblastic leukemia has been achieved mostly in serum and plasma specimens, a couple of relevant reports indicate their production by leukemic blasts in the bone marrow [44–47], supporting the notion that malignant cells directly contribute to the pathogenesis of ALL by creating an inflammatory microenvironment.

It remains to be seen whether the cytokine production in B-ALL results from a response to TLR ligation by pathogen-associated or damage-associated molecular patterns (PAMPs or DAMPs, resp.) [13, 37] or from a genetic aberration leading to a constitutive activation of inflammatory pathways [28]. As we have additionally found a significant production of inflammatory cytokines plus IL-6 by BM mesenchymal stromal cells (MSC) from B-ALL (unpublished data), we assume that some extrinsic stimuli may contribute to a bystander release of hematopoietic cytokines through activation of MSC [48].

**Figure 2:**
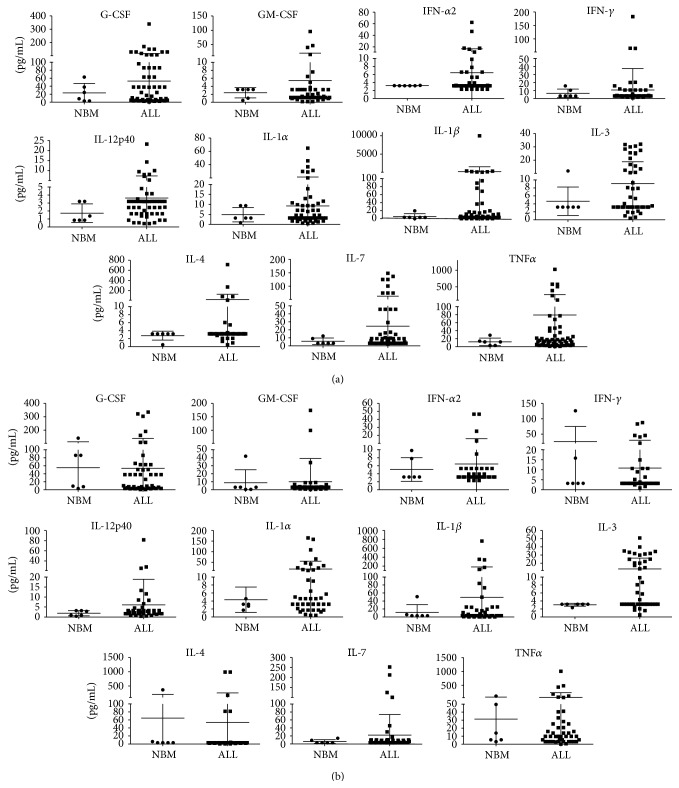
A distinct group of patients is highly producer of proinflammatory cytokines and hematopoietic growth factors. ALL or normal BM (NBM) mononuclear cells were cultured for 24 h (a) and supernatants immediately collected. Cells were provided with additional culture medium and supernatants collected 24 h later (b). The distribution of highly produced cytokines in NBM and ALL patients is shown.

Despite the heterogeneity in the abundance of the produced cytokines among patients, analyses of data dispersion indicated the existence of two groups, defined according to concentration of the produced cytokines upon 24 h culture (Figure 2(a)). Strikingly, a distinct and minor group behaved differently from most patients and from the normal controls, as they produced the highest amount of growth factors, interferons, and proinflammatory factors. Supernatants derived from this minor group and containing proinflammatory factors were designated as “inflammatory supernatants,” whereas supernatants containing low or normal amounts of proinflammatory factors were considered “noninflammatory supernatants.” Normal supernatants were derived from BM mononuclear cells from nonhematological individuals.

To explore the possibility of a local and transient cell preactivation, cell cultures were continued for additional 24 h upon the former supernatant collection. Concentration graphs in Figure 2(b) suggest a long-term activation of inflammatory pathways, independent on the continuous stromal cell contact within BM.

**Figure 3:**
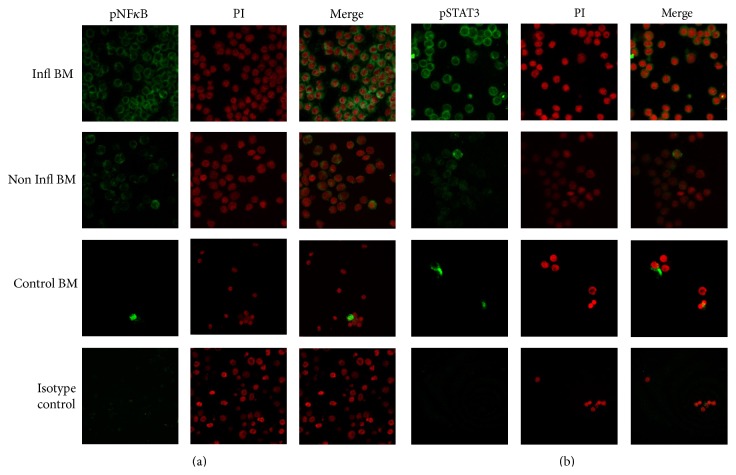
A proinflammatory profile is associated with activation of NF*κ*B and STAT3. Immunofluorescence microscopy was used to demonstrate pNF*κ*B p65 and pSTAT3 transcription factors within MNC fractions from ALL patients showing a proinflammatory profile (Infl BM) and a noninflammatory profile (Non Infl) or from normal individuals (Control BM). Propidium iodide was used for nuclear staining. A representative figure of four independent experiments is shown.

In order to investigate the pathways involved in the triggering of proinflammatory microenvironment, the activation status of p65 NF*κ*B and STAT3 was examined by indirect immunofluorescence (Figure 3). Among patients, the heterogeneity in activation levels of these transcription factors was a constant. However, samples showing a proinflammatory profile were mostly positive for phosphorylated NF*κ*B and STAT3, suggesting that, in this group of patients, the proinflammatory property is apparently mediated by the activation of NF*κ*B and STAT3 pathways (Figure 3, top panel). In contrast, BM samples identified as noninflammatory showed much less activation of both NF*κ*B and STAT3, and their normal counterpart shows scant basal activation. Interestingly, these transcription factors cooperate to promote the development and progression of cancer, and inhibitors of NF*κ*B and STAT3 have been proposed as therapeutically useful in switching the inflammatory nature of tumor environments. After activating their signaling pathways, the expression of antiapoptotic and proproliferative genes is under their control in both neoplastic and normal tissues [1, 49]. Moreover, studies indicate that leukemic stem cells in acute myelogenous leukemia have constitutively activated NF*κ*B [28], while STAT3 participates in the maintenance and self-renewal of HSC in TEL-AML1 t(12 : 21) ALL [50] and helps in the rebounding of the hematopoietic system through the control of myeloid progenitors differentiation [51].

### 3.2. The Inflammatory Profile Is Related to B-ALL BM Coexpressing Myeloid Markers

Among the 54 ALL patients included in this study, 34 fulfilled the criteria for high-risk disease by blood cell count, age, or Ph+ chromosome, whereas 17 fulfilled the criteria for standard-risk (Table 1). The median age values were 7.8 year old (2 mo–18 yr) and 4.8 (2 yr–9 yr) for the high-risk and standard-risk group, respectively. Independent of the conventional risk stratification, 20 patients showed aberrant coexpression of one or more myeloid markers, including CD13 and CD33.

Correlation tests were conducted by the Pearson method (not shown), showing weak or absent associations between risk stratification groups (Table 1) and the production of proinflammatory cytokines. No associations were found neither between single lymphoid markers (CD10, CD19, or CD20) or coexpressed single myeloid markers (CD13, CD14, CD15, or CD33) with production of particular cytokines. However, when the studied population was classified according to concomitant coexpression of 3 lymphoid markers (CD10, CD19, and CD20) or the aberrant expression of the myeloid markers CD13 and CD33, four groups were identified: a CD10+CD19+CD20+ positive group, which coexpressed these 3 B-lymphoid markers; a group lacking concomitant expression of CD10, CD19, and CD20, but expressing less than 3 of these B-lymphoid markers (CD10+/−CD19+/−CD20+/−) (Table 2); a CD13+CD33+ positive group; and a group expressing less than these 2 myeloid markers (Table 3). First, Mann-Whitney test was applied after the Kolmogorov-Smirnov analysis to discard normal distribution, and significant differences between the lymphoid positive (CD10+CD19+CD20+) and negative (CD10+/−CD19+/−CD20+/−) groups were found for IL-1*β*, TNF-*α*, G-CSF, and IFN-*α*2 with *P* ≤ 0.05 (Table 2). On the other hand, patients with aberrant coexpression of CD13 and CD33 in more than 25% of BM MNC were considered as a positive group in Table 3. Strikingly, *U* Mann-Whitney test showed significant differences between the two myeloid groups for IL-1*α*, IL-1*β*, IL-12p40, TNF-*α*, and GM-CSF (Table 3). Thus, aberrant expressions of myeloid markers within B-ALL BM apparently condition the abnormal production of proinflammatory factors. It remains to be investigated whether cells expressing myeloid markers constitute the origin of these factors, or they function as target and respond to proinflammatory factors by upregulating myeloid-related molecules. Alternatively, they might be newly differentiated under the emergency settings of ALL [42, 43, 48].

### 3.3. Early Progenitor Cells Are Driven into Cycle by Inflammatory Factors in B-ALL

**Figure 4:**
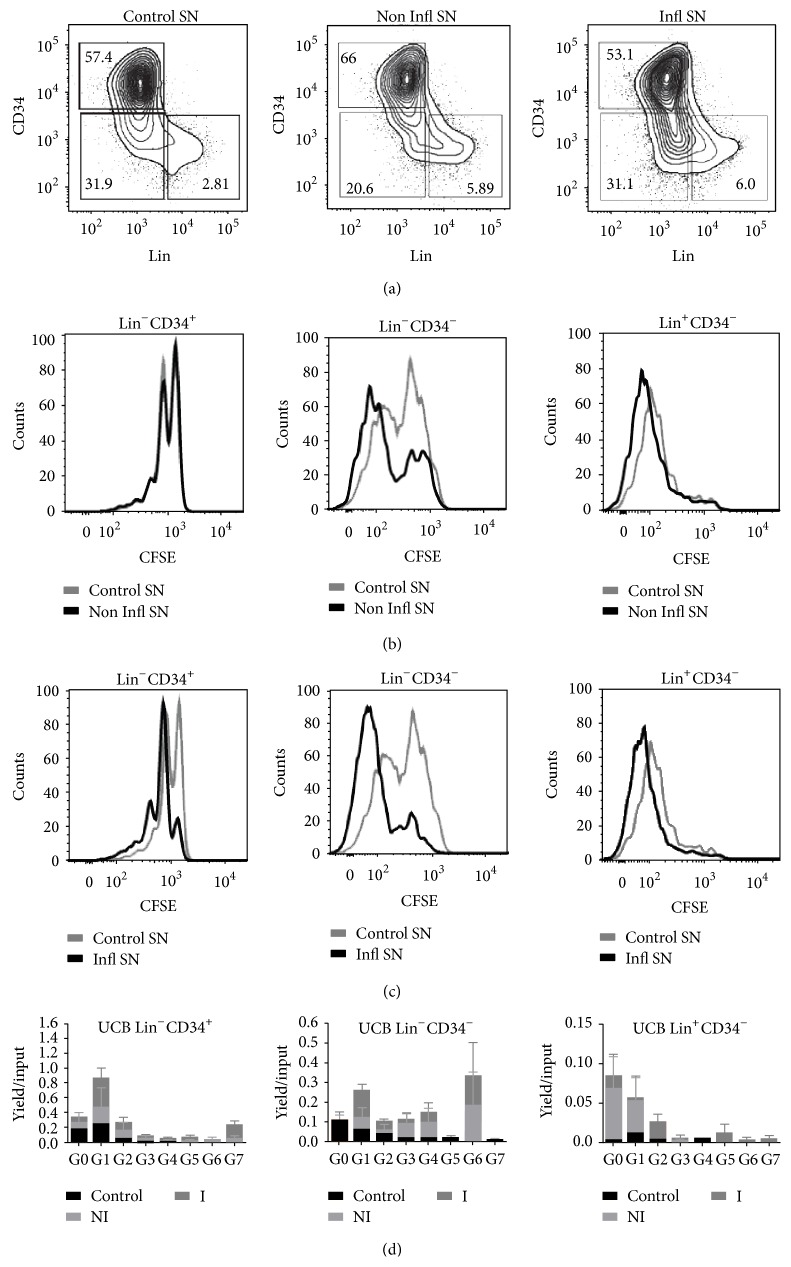
Proinflammatory factors produced by hematopoietic ALL BM cells promote the proliferation of normal primitive cells. Umbilical cord blood CD34^+^ cells were purified by magnetic separation and analyzed for their proliferation capabilities after 120 h exposure to control, noninflammatory, and inflammatory supernatants. The indicated gates were used to determine cell frequencies within the culture for Lineage^−^CD34^+^ primitive cells, Lineage^−^CD34^−^ differentiating precursor cells, and Lineage^+^CD34^−^ maturing cells (a) and to examine the number of cell divisions within each population. Exposure of cells to noninflammatory supernatants is shown in (b), whereas the inflammatory culture conditions are shown in (c). Total cell numbers within each cell generation and from each treatment condition were calculated and expressed as yields per input progenitor (d). Analyses of cell divisions (expressed as number of cell generations) were performed using the FlowJo software. Representative data of two independent experiments.

CFSE dilution assays were performed to test progenitor cell responses to inflammatory factors produced by the subset of cases of ALL expressing the myeloid antigens CD13 and CD33 within BM (Figure 4). Moderate acceleration of lineage commitment from Lin^−^CD34^+^ to Lin^+^CD34^−^ stages was recorded when multiparametric flow cytometry was used for phenotyping of the newly produced cells upon 5-day culture in lymphoid conditions (Figure 4(a)). In contrast, the induction to cell proliferation states by noninflammatory and inflammatory supernatants was clear (Figures 4(b) and 4(c)). While there was no apparent influence on the earliest cell compartment by noninflammatory factors, the differentiating Lin^−^CD34^−^ stage was selectively prompt to proliferate. Of special interest, inflammatory conditions clearly showed the awakening of primitive cells (Figure 4(c), left panel), as well as an intense activity on more differentiated Lin^−^CD34^−^ and Lin^+^CD34^−^ cells. The net positive effect was most appreciated when total absolute cell numbers of each particular stage were analyzed with respect to cell divisions (Figure 4(d)). Progenitor cells were driven into cycle by inflammatory conditions, as seen in the transition from cell generation 0 (G0) to generation 1 (G1) and downstream to generation 2 (G2). The promotion effect was also observed in differentiating Lin^−^CD34^−^ cells, where most cell generations from G1 to G6 contained mainly cells from the inflammatory condition (Figure 4, middle panel). In contrast, developing mature cells were restrictively promoted to proliferate up to G2 and in a similar extent as under noninflammatory conditions. According to these observations, previous reports indicate that during chronic inflammation, awakening of stem and progenitor cells leads to exhaustion of the pool [32, 34, 52]. Deciphering if the same is true for normal hematopoietic cells within ALL BM will be highly relevant and may help to predict risk or protective scenarios.

### 3.4. A Proinflammatory Environment of ALL BM Prompts Normal Hematopoietic Differentiation

**Figure 5:**
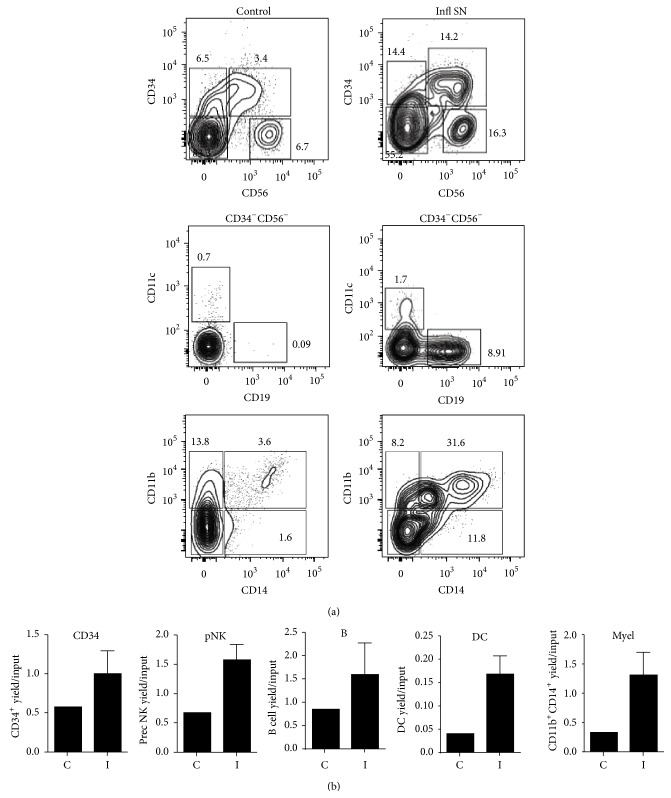
Early lymphoid- and myeloid-cell differentiation is substantially accelerated by inflammatory factors within ALL BM. CD34^+^ cells were purified from UCB and stimulated with control or inflammatory supernatants collected from normal or ALL BM, followed by a 3-week-stromal cell coculture. Newly produced cells were further identified and enumerated by multiparametric flow cytometry (a). The indicated gates were used to determine cell frequencies within the culture for CD34^+^ cells, CD34^+^CD56^+^ differentiating NK precursor cells, and CD56^+^ NK cells (shown in (a) upper panel). Further fractionation of the CD34^−^CD56^−^ compartment allows the identification of CD11c^+^ DC and CD19^+^ B cells ((a) middle panel). Myeloid cells appeared as CD11b^+^ or more mature CD11b^+^CD14^+^ cells ((a) lower panel). Total cell numbers from each treatment condition were calculated and expressed as yields per input progenitor (b). Control SN or C: control supernatant; Infl SN or I: inflammatory supernatant. Data are representative of three independent experiments. Supernatant from different individuals was used for each experiment.

To determine whether the ALL-inflammatory setting could influence early hematopoietic progenitor cell fate decisions, Lin^−^CD34^+^ cells from UCB were purified, exposed to normal or ALL supernatants, and cultured during 3 weeks under lymphoid conditions. Further cell harvesting revealed that CD34, NK, B, DC, and myeloid cell production were substantially increased by ALL-derived inflammatory supernatants when compared to normal controls. At the end of coculture, CB lymphoid progenitors showed at least twice yield per input progenitor upon incubation with ALL-factors (Figure 5). Of particular interest was the overproduction of innate immune NK and CD14^+^ myeloid cells, which resemble the stem and progenitor cell behavior under stress conditions or threatening infections [13, 32, 37, 43].

The function of some soluble factors is fundamental within the tumor microenvironment: TNF*α* potentiates growth of malignant skin, ovarian, and pancreatic tumors and is part of a network of proinflammatory molecules including CXCL12 and CCL2 chemokines, IL-6, macrophage inhibitory factor (MIF), and vascular endothelial growth factor (VEGF), among others [11]. For hematological neoplasias, there are experimental observations indicating the use of autocrine TNF*α* by leukemic initiating cells for their survival and proliferation, in a NF*κ*B-dependent manner [53]. On the other hand, the participation of IL-1*β* in the pathogenesis of acute myeloid leukemia has been documented, indicating that its signals in leukemic stem cells disrupt the dormant state and induce its proliferation potential [54]. Moreover, regulation of the NLRP3 inflammasome may explain the activity of IL1*β* in promyelocytic leukemia, as described in other models [55, 56]. The contribution of a particular inflammasome in the activation of inflammatory processes in B-ALL remains to be determined, as well as the putative activator of the oligomer inside the ALL cells.

To our knowledge, this is the first investigation on the influence of an inflammatory microenvironment in early hematopoietic development during lymphoid leukemia settings. The findings suggest that an ALL subset expressing myeloid antigens is associated with* in vitro* secretion of a number of cytokines, predominantly Th1-type, which, in turn, regulate proliferation and differentiation of primitive normal cells. Our data entails the NF*κ*B and STAT3 signaling in ALL cells with the creation of abnormal hematopoietic niches that may disrupt effective long-term blood cell formation processes. The mechanisms involved may include induction of growth factor receptors [37]. Of special interest will be the implication of specific genetic aberrations in the triggering of ALL-inflammation setting. Although a role for NF*κ*B in leukemogenesis is possible, the etiology and maintenance of ALL remain unknown in most cases [57]. The included patients in this study were not clinically identified as infected, arguing in favor of a “sterile” local inflammation. Besides, a conspicuous population of individuals behaved as “noninflammatory,” suggesting that the abnormal environment appears as a consequence of the activity of leukemic cells.

## 4. Concluding Remarks

There are great expectations for the biomedical research to unravel fundamental aspects of ALL pathogenesis from the earliest events of normal and neoplastic differentiation within bone marrow. Although the stability of the hematopoietic system has long been recognized, it has become clear that plasticity of stem and progenitor cells allows the crucial regulation of primitive cell compartments during inflammation. Furthermore, recent studies have highlighted the importance of chronic inflammation in development and maintenance of some malignancies. Our findings suggest the manipulation of the environment by a special type of leukemic cells that might disrupt the normal HSC-niche communication at the time tumor progression is promoted. The relevance of a rigorous multiparametric flow cytometry phenotyping and purification of the suggested leukemic cell populations producing proinflammatory cytokines is high for current and future investigations.

Decoding its possible relationship to the biology and prognosis of childhood acute leukemias is crucial for the design of therapeutic strategies based on manipulation of the tumor microenvironment.

## Figures and Tables

**Table 1:** Patient characteristics.

Patient	Age (yr)	Sex	WBC/mm^2^	Phenotype	Risk stratification Risk factor	Aberrant expression of myeloid markers	Concomitant expression of CD10/CD19/CD20
1	4	F	33700	B-ALL	SR	NEG	POS

2	4	F	7600	B-ALL	SR	CD15	POS

3	11	F	65000	B-ALL	HR Leukocytosis	NEG	POS

4	4	M	26400	B-ALL	SR	CD13/CD33	NEG

5	11	M	36600	B-ALL	HR Age	CD13/CD33	NEG

6	5	M	23000	B-ALL	HR PhCr+	CD13	POS

7	5	M	8900	T-ALL	HR T-ALL	NEG	POS

8	2	F	91500	B-ALL	HR Leukocytosis PhCr+	NEG	POS

9	1	M	40000	B-ALL	HR Age	NEG	NEG

10	10	F	1500	B-ALL	HR Age	NEG	POS

11	12	F	1300	B-ALL	HR Age	CD13/CD15	NEG

12	2	M	39000	B-ALL	SR	NEG	POS

13	5	M	11400	B-ALL	SR	NEG	NEG

14	13	M	3400	B-ALL	HR Age	NEG	NEG

15	5	M	1100	B-ALL	SR	CD13	NEG

16	2	M	5300	B-ALL	SR	NEG	NEG

17	3	F	260000	B-ALL	HR PhCr+	CD13	NEG

18	3	M	85700	B-ALL	HR Leukocytosis	CD13	NEG

19	4	M	161500	B-ALL	HR	NEG	POS

20	13	M	1600	M-ALL	HR	CD13/MPO	NEG

21	13	M	2600	B-ALL	HR Leukocytosis	NEG	NEG

22	3	M	400500	T-ALL	HR T-ALL	NEG	NEG

23	3	M	52500	B-ALL	HR Leukocytosis	CD-13	NEG

24	3	F	35100	B-ALL	SR	CD15	NEG

25	10	M	3500	B-ALL	SR	NEG	NEG

26	10	F	1200	B-ALL	HR Age	CD13/CD15	NEG

27	6	M	115000	B-ALL	HR Leukocytosis	CD13	NEG

28	2	M	—	B-ALL	—	NEG	POS

29	6	M	131000	B-ALL	HR Age	CD13	NEG

30	6	M	83500	B-ALL	HR Leukocytosis	NEG	POS

31	3	F	2700	B-ALL	SR	NEG	POS

32	6	M	13100	B-ALL	SR	NEG	POS

33	6	M	12600	B-ALL	SR	NEG	NEG

34	3	M	15600	B-ALL	SR	NEG	POS

35	4	F	—	B-ALL	—	NEG	NEG

36	6	M	1000	B-ALL	SR	NEG	NEG

37	14	F	600	B-ALL	HR PhCr+	NEG	NEG

38	14	M	163000	B-ALL	HR Leukocytosis	NEG	POS

39	10	M	5900	B-ALL	HR Age	CD13	POS

40	4	M	16100	B-ALL	HR PhCr+	NEG	NEG

41	15	M	113000	B-ALL	HR Age Leukocytosis	NEG	NEG

42	6	M	42100	B-ALL	SR	NEG	NEG

43	15	M	47400	B-ALL	HR Age	NEG	NEG

44	9	M	185000	B-ALL	HR Leukocytosis	NEG	POS

45	7	M	4000	B-ALL	SR	CD13	NEG

46	17	M	—	B-ALL	HR Age	NEG	NEG

47	0.9	F	334700	B-ALL	HR Age	CD33	POS

48	1	F	87900	B-ALL	HR Leukocytosis	NEG	POS

49	7	M	10500	B-ALL	SR	CD13/CD15	NEG

50	8	M	—	B-ALL	—	CD15	NEG

51	9	M	1900	B-ALL	HR	NEG	NEG

52	0.4	F	41600	B-ALL	HR Age	CD13	NEG

53	8	F	52500	B-ALL	HR Leukocytosis	NEG	NEG

54	8	F	350000	B-ALL	HR Leukocytosis	NEG	POS

yr: years old; WBC: white blood cell count; M: male; F: female; B-ALL: B-cell precursor acute lymphoblastic leukemia; T-ALL: T-cell acute lymphoblastic leukemia; M-ALL: mixed acute leukemia; HR: high risk; SR: standard risk.

**Table 2:** BM-expressing less than 3 B-ALL lymphoid markers produce high levels of proinflammatory factors.

Cytokine	Expression of 3 lymphoid markers (CD10+ CD19+ CD20+) Median (pg/mL) IR	Less than 3 positive lymphoid markers (CD10+/− CD19+/− CD20+/−) Median (pg/mL) IR	*P*
IL-1*α*	3.20 4.07	4.58 9.20	0.202

IL-1*β*	3.20 6.90	10.00 8.80	0.004^**^

IL-12p40	2.44 2.20	3.00 2.83	0.167

TNF*α*	9.85 13.72	19.00 54.73	0.032^*^

G-CSF	9.00 45.92	37.93 102.38	0.049^*^

GM-CSF	3.00 0.45	3.20 1.22	0.08

IL-7	5.00 6.39	9.18 42.51	0.104

IFN-*α*2	0.700 0.700	2.20 3.80	0.008^**^

^∗^ *P* = 0.05; ^∗∗^ *P* = 0.001.

Concomitant expression of CD10, CD19, and CD20. *U*-Mann Whitney test; IR: interquartile range; CMN: mononuclear cells.

**Table 3:** Aberrant expression of myeloid markers in B-ALL and production of proinflammatory factors.

Cytokine	Expression of the myeloid markers CD13 and CD33 Median (pg/mL) IR	Expression of less than two myeloid markers (CD13 or CD33) Median (pg/mL) IR	*P*
IL-1*α*	46.00 52.38	3.20 96.39	0.009^**^

IL-1*β*	429.00 9911.00	3.20 13.96	0.008^**^

IL-12p40	8.00 6.80	3.00 1.53	0.051^*^

TNF*α*	237.00 342.49	13.92 22.14	0.022^*^

G-CSF	37.00 104.17	24.32 81.19	0.558

GM-CSF	7.00 1.69	3.20 0.20	0.015^*^

IL-7	17.00 36.71	6.00 12.80	0.222

INF *α*2	8.00 12.80	3.20 3.80	0.122

^∗^ *P* = 0.05; ^∗∗^ *P* = 0.001.

Concomitant expression of CD13 and CD33. *U*-Mann Whitney test; IR: interquartile range.
